# Heritability and major gene effects on left ventricular mass in the Chinese population: a family study

**DOI:** 10.1186/1471-2261-6-37

**Published:** 2006-08-31

**Authors:** Kuo-Liong Chien, Hsiu-Ching Hsu, Ta-Chen Su, Ming-Fong Chen, Yuan-Teh Lee

**Affiliations:** 1 Institute of Preventive Medicine, School of Public Health, National Taiwan University, Taipei, Taiwan; 2 Department of Internal Medicine, National Taiwan University Hospital, Taipei, Taiwan

## Abstract

**Background:**

Genetic components controlling for echocardiographically determined left ventricular (LV) mass are still unclear in the Chinese population.

**Methods:**

We conducted a family study from the Chin-San community, Taiwan, and a total of 368 families, 1145 subjects, were recruited to undergo echocardiography to measure LV mass. Commingling analysis, familial correlation, and complex segregation analysis were applied to detect component distributions and the mode of inheritance.

**Results:**

The two-component distribution model was the best-fitting model to describe the distribution of LV mass. The highest familial correlation coefficients were mother-son (0.379, *P *< .0001) and father-son (0.356, *P *< .0001). Genetic heritability (h^2^) of LV mass was estimated as 0.268 ± 0.061 (*P *< .0001); it decreased to 0.153 ± 0.052 (*P *= .0009) after systolic blood pressure adjustment. Major gene effects with polygenic components were the best-fitting model to explain the inheritance mode of LV mass. The estimated allele frequency of the gene was 0.089.

**Conclusion:**

There were significant familial correlations, heritability and a major gene effect on LV mass in the population-based families.

## Background

Left ventricular (LV) hypertrophy profoundly affects morbidity and mortality from cardiovascular diseases, including myocardial infarction, congestive heart failure, and stroke [1,2]. Therefore, it is important to measure LV mass and manage LV hypertrophy among the hypertensive population in clinical practice [3]. The noninvasive imaging methods of echocardiography have greatly expanded our capability in evaluating increased LV mass, and have thus enhanced our understanding of the natural history of LV hypertrophy [4-6]. Blood pressure and volume overload are recognized as a strong determinant of LV hypertrophy [7]; however, interindividual variations in LV mass can be explained, only to a limited extent, by hemodynamic load [8]. Moreover, LV hypertrophy may occur in the absence of hypertension [9]. Genetic components have been considered as important factors for LV mass, and twin studies have shown that heritability accounts for up to 20–70% [10-13].

The family member study design is a useful tool for investigating genetic and environmental components of complex traits [14]. Significant heritability and evidence of strong familial aggregation in LV mass have been reported in different populations, such as in Caucasian [10,13,15-18], African Americans [17,19], American Indians [12] and Caribbean Hispanic families [20]. But data based on Chinese are not available. Furthermore, investigation on the mode of inheritance by segregation analysis among families can provide parametric estimates for linkage analyses [21]. Even though some major susceptibility genes have already been identified, segregation analysis can provide further evidence for major gene effects in the trait [22]. Previous segregation study showed the mode of inheritance of LV mass was compatible to polygenic model [13], but the study did not prove major gene effects controlling LV mass. Segregation analysis can investigate if the major gene effects exist, besides the polygenic background effects, and help to facilitate the further genomic study.

The study of young families from one community could provide a feasible way to investigate genetic components among the general population, because hypertension complications are comparatively rare and the environmental factors are more homogeneous than hospital-based subjects. The researchers in the Tecumseh Offspring Study showed that among the young population (mean 17 years old), parental LV mass explained a meaningful small percentage for adolescent offspring LV mass variance[23]. The community-based family study was to investigate the mode of inheritance of LV mass in the Taiwanese population.

## Methods

### Subjects

This family study was part of the Chin-Shan Community Cardiovascular Cohort Study, a prospective cohort study beginning in 1990 [24,25]. The family study arm started in 1997 and was designed to recruit adolescent probands from students in the only junior high school in the community. The proposal was approved by the Institutional Review Board of National Taiwan University Hospital and oral informed consent was obtained in each participant.

At first, a total of 1063 students (with a response rate of 94.6%) agreed to participate in a general health check-up after informed consent was obtained. They underwent examinations for anthropometric measures, blood pressure, lipid profiles and echocardiographic measures. The selected youths were stratified into two groups on the basis of seven measures, including total cholesterol, triglyceride, low density lipoprotein (LDL) cholesterol, body mass index, systolic pressure, diastolic pressure, and high density lipoprotein (HDL) cholesterol. There were 368 students selected for this study. All the students with values below the 10th percentile for HDL cholesterol and above the 90th percentile (for all students in this study) for any of the other six measures, were ascertained as high-risk probands (n = 171). The control young probands (n = 197) were randomly sampling from other young students. After obtaining informed consent from probands' family members, the same measures were performed for each family member.

Because the original stratification was not based exclusively on LV mass and the results of segregation analysis for the families were similar, the results of all families together with correction for ascertainment on proband's status were reported in this study. The ascertainment strategy is to select probands in upper 90^th ^percentiles of the risk factor distribution, and this strategy can substantially increase the power over random sampling. We used the proband high risk status as a surrogate strategy, which could identify individuals in high extreme LV mass distribution in the regressive model.

### Echocardiographic examination

Echocardiographic measures have been described in detail previously [4]. Four cardiologists performed and read the measurements. All subjects were asked to lie in the left lateral decubitus position to assure standardized measurement. We recorded the real-time image in video tapes simultaneously and calculated the measures in consecutive beats. We checked the agreement and reliability among these 4 cardiologists as follows. A sub-sample of 15 participants was selected to receive echocardiography by all four physicians to estimate the inter-observer variability of measurement parameters. The intraclass correlation reliability was calculated by a simple replication one-way analysis of variance test [26]. The values of interrater correlation coefficient reliability of LV mass were 0.80, indicating good agreement.

The LV mass was calculated by the Penn convention [27], and normalized LV mass values were calculated by one allometric value of height of power, 2.7, as suggested by de Simone et al [28].

### Statistical analysis

LV mass and related characteristics of the study participants were specified by proband, sibling and parent status. Mean, standard deviation, skewness and kurtosis were presented to show the distribution of related traits. The residual LV mass from the multiple linear regression model, after adjusting for age, gender, body mass index, smoking, and alcohol drinking status, plus sample mean, were used for further genetic analysis.

### Genetic analysis

We used commingling analysis on LV mass values to test whether the data were best described by one, two, or three more Normal distributions by ADMIX program [29]. The parameters for each component's mean, variance and proportion were estimated by the maximum likelihood method. The best-fitted commingling distribution model was defined by comparing the likelihood ratio test statistics among different nested models.

The intra-familial correlation coefficients of LV mass trait were measured in different pairs, including spouse, parent-offspring, and siblings, by FCOR program in S.A.G.E. [30]. We tested if the correlation coefficients of parent-offspring pairs were statistically different from zero by the Fisher's z test [31]. Heritability estimate of LV mass in the families was estimated by the variance component model, which was implemented in the SOLAR software [32].

### Complex segregation analysis

Segregation analysis of adjusted LV mass was conducted using regressive models as implemented in the REGC program in S.A.G.E. [30]. These models assume that the variation of LV mass among family members is the result of a major gene effect, with residual variation reflecting both familial correlations and individual variation. The presence of a major gene is assessed by allowing two factors or alleles (A and B) at a single locus, resulting in three 'ousiotypes' (AA, AB, BB) in individuals. The means of LV mass for each ousiotype is denoted μ_AA_, μ_AB_, μ_BB_, with one common variance of σ^2^. The frequencies of allele A and B are denoted q_A _and (1- q_A_), respectively. The distribution of types in the population is assumed to be in Hardy-Weinberg equilibrium. Individuals of each type are assumed to transmit allele A to their offspring with transmission probabilities τ_AA_, τ_AB _and τ_BB_, respectively. Residual familial resemblance unable to be explained by this major locus is modeled by familial correlations. The correlation between spouses, parents and offspring, mother and offspring, father and offspring, and between siblings are denoted ρ_MF_, ρ_PO_, ρ_MO_, ρ_FO_, and ρ_SS_, respectively. For this study, we adopted class D regressive models, in which residual sib-sib correlations are equal among all sibs of common parentage and can be due to any cause. If ρ_PO _is held equal to ρ_SS_, these models have been shown to be mathematically and numerically equivalent to the conventional mixed model of inheritance in nuclear families [33].

The analyses started with fitting a general model, in which all parameters were allowed to be estimated. Then we compared the general model with various submodels, in which certain parameters were restricted to specific values. Under a Mendelian model, the transmission probabilities, i.e., τ_AA_, τ_AB _and τ_BB_, were held equal to Mendelian expectations of 1, 0.5, and 0. A nontransmitted environmental effect model predicted that the probability of an individual having one ousiotype or another was independent of both the person's generation and the ousiotypes of his/her parents. For the environmental model in this study, each of the transmission probabilities was taken to be equal to the factor frequency, i.e., τ_AA _= τ_AB _= τ_BB _= q_A_. Both the Mendelian and environmental models can allow for residual familial correlations. A pure polygenic model assumed no major gene effect, so gene frequency and transmission probabilities were all fixed to one. The fit of hierarchical models was compared with the likelihood ratio test, calculated as -2 of the difference between the *ln *likelihood of the models being compared. The likelihood ratio value follows a chi-square distribution, with degrees of freedom equal to the difference between the models in the number of parameters estimated. Among nonhierarchical models, the most parsimonious model is that with the lowest values of Akaike's information criterion (AIC = -2 ln likelihood +2 [number of estimated parameters]) [34].

We used the adjusted LV mass values without a logarithm transformation for the segregation analysis since a normalizing transformation of a skewed trait would decrease the power to detect a major gene effect when one exists [35]. We fitted the environmental model to detect possible environmental effects. If such a model was rejected, the major gene effects were not caused by the skewness of the LV mass levels [36].

## Results

### Description of study participants

The characteristics of LV mass and related factors among probands, siblings, and their parents are presented in Table 1. The parents had higher blood pressure, LV mass, and smoking and drinking rates than the probands and siblings. In addition, the parents had larger standard deviations in BMI, blood pressure and LV mass measures than their offspring. The values of skewness and kurtosis showed that LV mass measures were nearly zero, indicating nearly normal distribution in LV mass. The proportion of the variation in LV mass in this sample explained by gender, age, body mass index, smoking and drinking status was 51%.

**Table 1:** Basic characteristics of participants in the study, specified by generations (n = 1,145)

	Probands (N = 368)				Siblings (N = 333)				Parents (N = 444)			
			
	Mean	SD	Skewness	Kurtosis	Mean	SD	Skewness	Kurtosis	Mean	SD	Skewness	Kurtosis
Age (years)	16.45	0.97	0.33	0.34	17.83	3.43	0.59	1.05	43.65	5.74	1.35	4.65
BMI (kg/m^2^)	20.63	3.87	1.13	1.24	20.08	3.13	1.31	4.26	24.23	3.91	1.03	4.40
SBP (mmHg)	108.86	13.02	0.34	-0.34	111.65	12.90	0.36	0.27	119.13	15.12	0.82	1.28
DBP (mmHg)	67.79	9.33	-0.05	-0.75	70.66	10.36	0.18	0.51	78.48	11.46	0.70	0.68
LV mass (gm)	117.02	39.99	1.00	1.06	111.10	38.26	0.91	0.87	150.08	48.65	0.62	0.21
LV mass index (gm/m^2.7^)	31.93	9.41	1.02	1.88	30.39	8.80	0.87	1.49	41.16	11.59	0.47	-0.11
												
Smoking	1 (0.3%)				35 (10.5%)				159 (35.8%)			
Alcohol drinking	1 (0.3%)				25 (7.5%)				173 (39.0%)			

Abbreviated: BMI: body mass index, SBP: systolic blood pressure, DBP: diastolic blood pressure, LV: left ventricular

### Commingling analysis results

**Figure 1:**
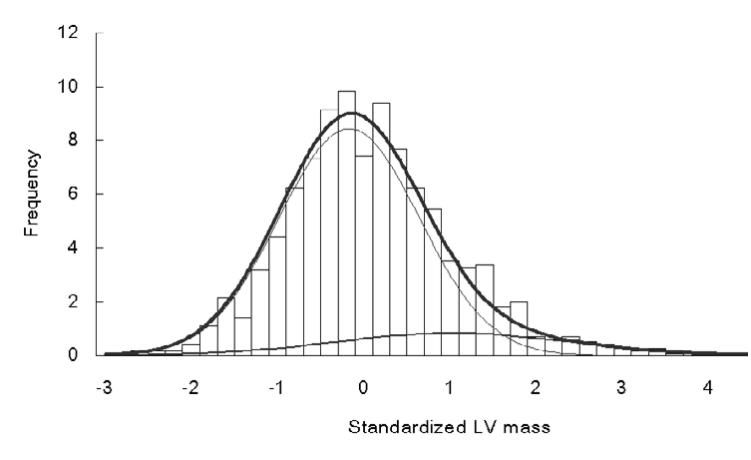
Observed (histogram) and expected (linear) distributions of adjusted left ventricular mass in the study subjects. Two thin lines were plotted from 2-component Normal distributions with means as (-0.168, 1.029) and variance as (0.661, 1.837), respectively. The thick line was the summation of two thin lines.

Commingling analysis showed that a 2-component, rather than a single-component distribution, was the best-fit model for LV mass variations. The component means, variances, and proportions for the 2-component distribution model were (-0.168, 1.029), (0.661, 1.837) and (85.9%, 14.1%), respectively. The χ^2 ^test for comparing the 2-component with the 3-component distribution was not significant (χ^2 ^= 5.45, df = 3, p = 0.142), indicating that the 2-component distribution was the best-fit model. It implied that there were major gene effects. The observed and expected distributions from commingling results were plotted in Figure 1. This shows close approximation of expected to observed distribution.

### Familial correlation and genetic heritability estimates

Significant spouse, parent-offspring and sibling coefficients were found (Table 2), and the highest values were mother-son (0.379, *P *< .0001) and father-son (0.356, *P *< .0001). There were significant spouse and sibling correlation coefficients (0.254, 0.169, both *P *< .0001). Genetic heritability (h^2^) of LV mass was estimated as 0.268 ± 0.061 (*P *< .0001), indicating significant genetic components controlling LV mass trait. After adjusting for systolic pressure, heritability decreased to 0.153 ± 0.052 (*P *= .0009).

**Table 2:** Familial correlation coefficients and significant levels for testing the null hypothesis among the various familial pairs in the studied family members.

Pairs	Numbers	Coefficients	*P *value
Spouse	162	0.254	0.001
Parent-offspring	889	0.294	0.000
Sibling	427	0.169	0.000
Grandparent-child	287	0.179	0.002
Mother-daughter	220	0.254	0.000
Mother-son	252	0.379	0.000
Father-daughter	205	0.084	0.231
Father-son	212	0.356	0.000
Sister-sister	131	0.136	0.121
Sister-brother	209	0.162	0.019
Brother-brother	87	0.208	0.053

### Complex segregation analysis result

We used the complex segregation analysis to detect if major gene effects exist to control LV mass trait. We constructed several models, including sporadic, polygenic, environmental and major gene (Mendel) models to estimate the parameters such as gene frequency, transmission, penetrance and correlations from family data (Table 3). Compared with a general unrestricted model, the chi-square and corresponding *P *values were presented. We found that the pure major gene effects model and mixed codominant model were similar with the full model (*P *values 0.370 and 0.214, respectively). The pure major gene effects model had the smallest AIC value and was considered as the best-fit model to explain the mode of inheritance of LV mass. Under this major gene effects model, we found that the gene, which controlled high LV mass values, had an allele frequency of 0.098, and the three means of LV mass were 64.4, 45.7 and 33.9 gm/m. Polygenic effects, presented by familial correlation, were also demonstrated and there was significant spouse correlation up to 0.327.

**Table 3:** Parameter estimates from segregation analysis of residual LV mass values: Class D regressive models, conditional on proband phenotypes

Model	Pure familial correlation, i.e. polygenic		Pure major gene (Mendel)		Mendel + familial correlation (mixed codominant)		A Dominant, μ_AB _= μ_AA_		A Recessive, i.e. B Dominant, μ_AB _= μ_BB_		Unrestricted, general	
						
	Parameter	S.E.	Parameter	S.E.	Parameter	S.E.	Parameter	S.E.	Parameter	S.E.	Parameter	S.E.
qA	[1]		0.098	0.028	0.093	0.030	0.040	0.020	0.000	0.142	0.109	0.024
τ_AA_	-		[1]		[1]		[1]		[1]		1.000	0.000
τ_AB_	-		[0.5]		[0.5]		[1]		[1]		0.249	0.100
τ_BB_	-		[0]		[0]		[0]		[0]		0.034	0.030
μ_AA_	36.0	0.5	64.4	4.3	65.1	4.5	51.4	2.8	40.9	8.2	62.7	3.4
μ_AB_	= μ_AA_		45.6	1.8	45.7	1.9	51.4	2.8	36.0	0.5	46.1	1.4
μ_BB_	= μ_AA_		33.7	0.5	33.9	0.6	34.9	0.6	36.0	0.5	33.5	0.6
σ^2^	80.6	4.6	52.4	4.2	53.5	4.7	61.8	5.5	80.6	4.6	47.1	4.9
ρ_MF_	0.199	0.072	0.327	0.093	0.315	0.091	0.246	0.079	0.199	0.072	0.334	0.098
ρ_MO_	0.196	0.050	[0]		0.058	0.070	0.130	0.067	0.196	0.050	0.076	0.079
ρ_FO_	0.093	0.059	[0]		-0.070	0.069	-0.025	0.064	0.093	0.059	-0.051	0.076
ρ_SS_	0.118	0.055	[0]		0.000	0.000	0.037	0.069	0.118	0.055	0.017	0.065
# of	6		6		9		8		8		12	
parameters												
-2ln(L)	4721.80		4684.40		4682.38		4688.22		4721.80		4677.90	
AIC	4733.8		4696.4		4700.38		4704.22		4737.8		4701.9	
χ^2^	43.9		6.5		4.48		10.32		43.9			
*P*	0.000		0.370		0.214		0.035		0.000		**Baseline**	

Abbreviation: qA, allele frequency; τ_AA_, τ_AB_, τ_BB_, transmission probability; μ_AA_, μ_AB_, μ_BB_, genotype means; σ^2^, variance; ρ_MF_, ρ_MO_, ρ_FO_, ρ_SS_, correlation coefficients among spouse, mother-offspring, father-ffspring and sibling pairs; #, number of parameters; ln(L), logarithm of likelihood; AIC, Akaike information criteria; *P*, p-value by compared with the unrestricted general model.

## Discussion

Significant heritability and familial correlations of LV mass were proven in this study, based on a large number of adolescent proband families. We also demonstrated that the inheritance mode of LV mass was compatible with major genes effects among the Chinese population. This study had two scientific contributions. First, there is considerable homogeneity in this study population. Most of the subjects live in the same community; hence their social and living environments tended to be more similar than those in different communities. Also, the characteristics of study subjects can avoid the potential bias of recruitment from a hospital setting. Second, the results are particularly relevant for a population at low risk for atherosclerosis, since the probands were systematically selected from young adolescents in the community, in which the complication of hypertension and LV hypertrophy were not prevalent in the study population.

There are many reports on left ventricular mass heritability with estimates from different populations (Table 4). Most studies showed significant heritability and sibling correlations, especially among African Americans. Our study results were compatible with Caucasian population studies [13,15,17]. The high heritability estimates in African Americans might be due to ascertainment from hypertensive hyperlipidemic proband siblings [19]. Caribbean Hispanics population had high estimate [20]. Variation on heritability estimates of LV mass might be explained by different ethnicity. Also, the different expression of a complex polygenic trait in relation to unmeasured factors might be involved.

**Table 4:** Summary of heritability estimates of left ventricular mass trait

**Author, year**	**Study design**	**Study population origins**	**Numbers of families/subjects**	**Adjusted covariate**	**Heritability estimate (h^2^)**	**Comments**
Post et al. 1997 ^15^	Relative pairs	Caucasians, Framingham families, no systemic diseases	2624 subjects	Age, height, weight, systolic blood pressure	0.24–0.32	Limit in subjects without systemic diseases nor medication history
Garner et al. 2000 ^13^	Nuclear families	White, European families	149/624	weight	0.28	Children population
Kotchen et al. 2000 ^19^	Sibling	African American, hypertensive, hyperlipidemia	68 sibpairs	Age, gender	0.65–0.72	FCOR and ASSOC in SAGE program to perform the analysis
Arnett et al. 2001 ^16^	Sibling	African Americans & white hypertensive	1664 siblings	Age, gender, systolic blood pressure, obesity	Sibling correlations 0.29–0.44 in African American, 0.04–0.12 in white	High sibling correlation in African Americans
Palatini et al. 2001 ^23^	Nuclear families	Tecumseh Offspring Study	251 offspring and 290 parents	Age, gender, body height systolic blood pressure, insulin, urinary sodium excretion,	No estimate of h^2^, parental LV mass explained 7.6% of total variance of offspring LV mass	Multiple linear regression models Young offspring (mean 17 years old)
Mayosi et al. 2002 ^17^	Extended families	British Caucasians, hypertensive probands from hospital or clinics	229/955	Age, systolic blood pressure, weight, height, WHR, diabetes	0.23–0.29	Ascertainment correction: yes
Swan et al. 2003 ^18^	Twins	Caucasians, Population-based	55 pairs MZ vs. 55 pairs DZ	Age, gender, blood pressure, weight	0.53–0.69	
Bella et al. 2004 ^12^	Relative pairs, mostly sibpairs	American Indian families, different geographic location	455/1373, 1305 relative pairs, 1077 sibpairs	Age, gender, centers, weight, height, systolic blood pressure, heart rate, medication, diabetes	0.17(multiple adjusted) -0.27 (first three variates adjusted)	No significant heritability in Arizona Indians
Juo et al. 2005 ^20^	Extended families	Caribbean Hispanics	84/623	Age, gender, weight	0.51	Adding systolic blood pressure, diabetes, medication did not affect the estimate
This report	Nuclear families	Ethnic Chinese, young probands, community-based	368/1145	Age, gender, body mass index, blood pressure	0.15 (multiple adjusted) -0.27 (first 3 variates adjusted)	

The heritability decreased after adjusting for blood pressure. It implied possible pleiotrophic effects of genes on controlling blood pressure and LV mass. Hemodynamic load, such as stroke volume, has an influence on LV mass among young adults and adolescents, and this impact is more important than body size. However, high proportions of LV mass variations still remain unexplained [8]. Genetic components played important role in residual LV mass variations. We ascertained young proband families, where hypertension complications are comparatively rare, and from one community population, where environmental factors are rather homogeneous for genetic studies. Our study subjects were rather young, and our results showed results similar to the Tecumseh offspring study [23].

We found the highest correlations were in parent-son pairs, which indicated male offspring had influential effects from parents. Our estimate of sibling correlation was as the same as Framingham sibling pairs (0.16), while the estimates of parent-offspring and spouse correlations (0.29 and 0.25, respectively) were much higher than those in Framingham pairs (0.15 in parent-offspring, 0.05 in spouse) [15]. For intra-familial resemblance, greater father-offspring correlations were observed among the British Caucasian population, much higher than mother-child correlations [17]. Our study also showed sex-specific patterns of familial correlations; the highest was the parent-son pairs (0.36–0.38) and the lowest was the father-daughter (0.08). Different parent-of-origin effects on offspring LV mass was reported in European families [37]. The mother-offspring correlation coefficient was significantly higher than father-offspring correlation among the European population. The discrepancy implies the importance of ethnic difference on parental factors on offspring traits. Also, high spouse correlation implies that the common household effects were important for controlling LV mass. Lifestyle habits, such as salt intake and physical activity, might be also familial aggregation and may thus explain the high correlation between spouse pairs.

There were several studies on genomic profiles for LV mass. For example, a cross-breeding hypertensive rat model demonstrated two loci with high LOD scores [38,39]. There were many reports on candidate genes such as G-protein beta-3, aldosterone synthase, and beta-1 adrenoceptor genes associated with LV mass [18,40]. Our study results can provide further genomic research on LV mass.

### Study limitations

The limitations of our study were as follows. Firstly, only mathematical modeling methods such as commingling and segregation analyses were investigated and no candidate gene markers were investigated. Although we postulated one major gene with allele frequency around 0.1 controlling LV mass and 2-component commingling patterns, we did not shed light on which genes most likely involved. Secondly, epistasis among genes or gene-environmental interaction cannot be explored in this study. Incorporation with environmental factors can elucidate the possible roles of risk factors and interaction effects.

## Conclusion

We showed that there were significant parent-son correlation coefficients, genetic heritability and major gene effects controlling LV mass among ethnic Chinese in Taiwan. Candidate gene markers could be used to investigate the association and linkage with LV mass.

## Competing interests

The author(s) declare that they have no competing interests.

## Authors' contributions

KLC carried out the data collection, statistical analyses, participated in the study design and processing data. YTL participated in the design of the study and supervised the ideas developed in hypothesis generation. MFC & HCH performed the laboratory measurement in lipid levels and were in charge of quality control. TCS carried out the data collection. All authors read and approved the final manuscript.

## Pre-publication history

The pre-publication history for this paper can be accessed here:



## References

[B1] Levy D (1988). Left ventricular hypertrophy epidemiological insights from the Framingham heart study. Drugs.

[B2] Kannel WB (2000). Fifty years of Framingham Study contributions to understanding hypertension. J Hum Hypertens.

[B3] Okin PM, Devereux RB, Jern S, Kjeldsen SE, Julius S, Nieminen MS, Snapinn S, Harris KE, Aurup P, Edelman JM, Wedel H, Lindholm LH, Dahlof B, LIFE Study Investigators (2004). Regression of electrocardiographic left ventricular hypertrophy during antihypertensive treatment and the prediction of major cardiovascular events. JAMA.

[B4] Chien KL, Sung FC, Hsu HC, Su TC, Lee YT (2001). Left Ventricular mass and correlated atherosclerotic risk factors in young adolescents: report from Chin-Shan community cardiovascular study in Taiwan. Atherosclerosis.

[B5] Devereux RB, Roman MJ, de Simone G, O'Grady MJ, Paranicas M, Yeh JL, Fabsitz RR, Howard BV (1997). Relations of left ventricular mass to demographic and hemodynamic variables in American Indians: the Strong Heart Study. Circulation.

[B6] Goble MM, Mosteller M, Moskowitz WB, Schieken RM (1992). Sex differences in the determinants of left ventricular mass in childhood. The Medical College of Virginia twin study. Circulation.

[B7] Ganau A, Devereux RB, Pickering TG, Roman MJ, Schnall PL, Santucci S, Spitzer MC, Laragh JH (1990). Relation of left ventricular hemodynamic load and contractile performance to left ventricular mass in hypertension. Circulation.

[B8] de Simone G, Devereux RB, Kimball TR, Mureddu GF, Roman MJ, Contaldo F, Daniels SR (1998). Interaction between body size and cardiac workload: influence on left ventricular mass during body growth and adulthood. Hypertension.

[B9] Chen CH, Ting CT, Lin SJ, Hsu TL, Ho SJ, Chou P, Chang MS, O'Connor F, Spurgeon H, Lakatta E, Yin FC (1998). Which arterial and cardiac parameters best predict left ventricular mass?. Circulation.

[B10] Bielen E, Fagard R, Amery A (1991). The inheritance of left ventricular structure and function assessed by imaging and Doppler echocardiography. Am Heart J.

[B11] Busjahn A, Knoblauch H, Knoblauch M, Bohlender J, Menz M, Faulhaber HD, Becker A, Schuster H, Luft FC (1997). Angiotensin-converting enzyme and angiotensinogen gene polymorphisms, plasma levels, cardiac dimensions. A twin study. Hypertension.

[B12] Bella JN, MacCluer JW, Roman MJ, Almasy L, North KE, Best LG, Lee ET, Fabsitz RR, Howard BV, Devereux RB (2004). Heritability of left ventricular dimensions and mass in American Indians: The Strong Heart Study. J Hypertens.

[B13] Garner C, Lecomte E, Visvikis S, Abergel E, Lathrop M, Soubrier F (2000). Genetic and environmental influences on left ventricular mass. A family study. Hypertension.

[B14] Khoury MJ, Beaty TH, Cohen BH (1993). Fundamentals of Genetic Epidemiology.

[B15] Post WS, Larson MG, Myers RH, Galderisi M, Levy D (1997). Heritability of left ventricular mass: the Framingham heart study. Hypertension.

[B16] Arnett DK, Hong Y, Bella JN, Oberman A, Kitzman DW, Hopkins PN, Rao DC, Devereux RB (2001). Sibling correlation of left ventricular mass and geometry in hypertensive African Americans and whites: the HyperGEN study. Hypertension Genetic Epidemiology Network. Am J Hypertens.

[B17] Mayosi BM, Keavney B, Kardos A, Davies CH, Ratcliffe PJ, Farrall M, Watkins H (2002). Electrocardiographic measures of left ventricular hypertrophy show greater heritability than echocardiographic left ventricular mass. Eur Heart J.

[B18] Swan L, Birnie DH, Padmanabhan S, Inglis G, Connell JM, Hillis WS (2003). The genetic determination of left ventricular mass in healthy adults. Eur Heart J.

[B19] Kotchen TA, Kotchen JM, Grim CE, George V, Kaldunski ML, Cowley AW, Hamet P, Chelius TH (2000). Genetic determinants of hypertension: identification of candidate phenotypes. Hypertension.

[B20] Juo SH, Di Tullio MR, Lin HF, Rundek T, Boden-Albala B, Homma S, Sacco RL (2005). Heritability of left ventricular mass and other morphologic variables in Caribbean Hispanic subjects: the Northern Manhattan Family Study. J Am Coll Cardiol.

[B21] Jarvik GP (1998). Complex segregation analyses: uses and limitations. Am J Hum Genet.

[B22] Cui J, Antoniou AC, Dite GS, Southey MC, Venter DJ, Easton DF, Giles GG, McCredie MR, Hopper JL (2001). After BRCA1 and BRCA2-what is next? multifactorial segregation analyses of three-generation, population-based Australian families affected by female breast cancer. Am J Hum Genet.

[B23] Palatini P, Krause L, Amerena J, Nesbitt S, Majahalme S, Tikhonoff V, Valentini M, Julius S (2001). Genetic contribution to the variance in left ventricular mass: the Tecumseh Offspring Study. J Hypertens.

[B24] Chien KL, Hsu HC, Su TC, Lee YT (2003). Consistency in genetic inheritance mode and heritability patterns of triglyceride vs. high density lipoprotein cholesterol ratio in two Taiwanese family samples. BMC Genet.

[B25] Chien KL, Chen WJ, Hsu HC, Su TC, Chen MF, Lee YT (2005). Major gene effects on apolipoprotein B concentrations in families of adolescents-results from a community-based study in Taiwan. Clin Chim Acta.

[B26] Fleiss JL (1986). The Design and Analysis of Clinical Experiments.

[B27] Devereux RB, Reichek N (1977). Echocardiographic determination of left ventricular mass in man anatomic validation of the method. Circulation.

[B28] de Simone G, Daniels SR, Devereux RB, Meyer RA, Roman MJ, de Divitiis O, Alderman MH (1992). Left ventricular mass and body size in normotensive children and adults: assessment of allometric relations and impact of overweight. JACC.

[B29] Cloninger CR, von Knorring L, Oreland L (1985). Pentametric distribution of platelet monoamine oxidase activity. Psychiatry Research.

[B30] (2006). S.A.G.E. 5.2, Statistical Analysis for Genetic Epidemiology. http://darwin.cwru.edu/sage/.

[B31] Snedecor GW, Cochran WG (1980). Statistical methods.

[B32] Almasy L, Blangero J (1998). Multipoint quantitative-trait linkage analysis in general pedigrees. Am J Hum Genet.

[B33] Demenais FM, Bonney GE (1989). Equivalence of the mixed and regressive models for genetic analysis. I Continuous traits Genetic Epidemiol.

[B34] Akaike H (1974). A new look at the statistical model identification. IEEE Transactions on Automatic Control.

[B35] Prenger VL, Beaty TH, Kwiterovich PO (1992). Genetic determination of high-density lipoprotein-cholesterol and apolipoprotein A-1 plasma levels in a family study of cardiac catheterization patients. Am J Hum Genet.

[B36] Demenais F, Lathrop M, Lalouel JM (1986). Robustness and power of the unified model in the analysis of quantitative measurements. Am J Hum Genet.

[B37] Kuznetsova T, Staessen JA, Olszanecka A, Ryabikov A, Stolarz K, Malyutina S, Fagard R, Kawecka-Jaszcz K, Nikitin Y, European Project On Genes in Hypertension (EPOGH) Investigators (2003). Maternal and paternal influences on left ventricular mass of offspring. Hypertension.

[B38] Innes BA, McLaughlin MG, Kapuscinski MK, Jacob HJ, Harrap SB (1998). Independent genetic susceptibility to cardiac hypertrophy in inherited hypertension. Hypertension.

[B39] Tsujita Y, Iwai N, Tamaki S, Nakamura Y, Nishimura M, Kinoshita M (2000). Genetic mapping of quantitative trait loci influencing left ventricular mass in rats. Am J Physiol Heart Circ Physiol.

[B40] Patel DA, Li S, Chen W, Srinivasan SR, Boerwinkle E, Berenson GS (2005). G-6A polymorphism of the angiotensinogen gene and its association with left ventricular mass in asymptomatic young adults from a biethnic community: the Bogalusa Heart Study. Am J Hypertens.

